# *FGFR3* Alterations in the Era of Immunotherapy for Urothelial Bladder Cancer

**DOI:** 10.3389/fimmu.2020.575258

**Published:** 2020-11-05

**Authors:** Alec Kacew, Randy F. Sweis

**Affiliations:** ^1^ Section of Hematology and Oncology, Department of Medicine, The University of Chicago, Chicago, IL, United States; ^2^ Committee on Immunology, The University of Chicago, Chicago, IL, United States; ^3^ Comprehensive Cancer Center, The University of Chicago, Chicago, IL, United States

**Keywords:** bladder cancer, fibroblast growth factor receptor, immunotherapy, pharmacogenetics, targeted molecular therapy

## Abstract

*FGFR3* is a prognostic and predictive marker and is a validated therapeutic target in urothelial bladder cancer. Its utility as a marker and target in the context of immunotherapy is incompletely understood. We review the role of *FGFR3* in bladder cancer and discuss preclinical and clinical clues of its effectiveness as a patient selection factor and therapeutic target in the era of immunotherapy.

## Introduction

Cytotoxic chemotherapy had been the only standard-of-care treatment for advanced urothelial bladder cancer, which is the world’s 10^th^ most common cancer and thirteenth most deadly (1). Cisplatin-based regimens are associated with objective responses in up to 45% of patients, but these responses are generally not durable (2, 3). Cisplatin-based therapies are associated with toxicities, including treatment-related mortality in rare cases. Beginning with the regulatory approval of atezolizumab, an inhibitor of programmed death-ligand 1 (PD-L1), in 2016, a total of five immune checkpoint inhibitors (ICIs), including the programmed cell death protein 1 (PD-1) inhibitors pembrolizumab and nivolumab and the PD-L1 inhibitors avelumab and durvalumab, gained regulatory approval for advanced urothelial cancer. These therapies are associated with durable responses in a minority of patients (roughly 15% among patients selected based on immune infiltration) and comparatively favorable side effect profiles (4). They have now been used in the first line alone and in combination with chemotherapy and are the preferred choice in the second line after chemotherapy (5–7).

In spite of the great therapeutic potential of ICIs, only a minority (approximately 20%) of patients experience tumoral response to ICIs and median survival with second line immunotherapy remains shorter than 1 year (8). It follows that the identification of biomarkers is a critical step in improving therapy for advanced urothelial bladder cancer. Recognition of characteristics associated with ICI response can help clinicians and researchers optimize patient selection, appreciate new combination or sequencing strategies, and identify mechanisms or targets for development of novel therapeutics. Tumoral PD-L1 expression is only modestly useful as a marker, as tumoral responses to ICI have been observed regardless of PD-L1 status (albeit at a numerically higher rate among those with greater PD-L1 expression) (9). Consensus molecular classifications, which define luminal, basal/squamous, stroma-rich, and neuroendocrine-like subgroups of muscle-invasive bladder cancer, although useful in understanding the biology of tumors, similarly fall short in helping to guide ICI therapy (10). The goal remains to discover tumor characteristics, drivers, and markers that can offer greater therapeutic and instructive value in the context of ICI therapy. Overactivity in the *ErbB* family (including *EGFR* and *Her2/neu*), which is associated with luminal and basal/squamous classifications, has only demonstrated utility as a drug target or predictive marker in a small proportion of clinical trials related to that pathway (11). Similarly, although *VEGF* activation portends poor outcomes, *VEGF* has not proved to be particularly promising as a therapeutic target (11). Mutations in DNA damage response genes, including *ERCC1, ERCC2, ATM, FANCC*, and *RB1* can help predict response to platinum-based therapy, but markers for newer immune-based therapies are needed (11). The fibroblast growth factor receptor 3 (*FGFR3*) gene has long been associated with bladder cancer oncogenesis and recently become a therapeutic target (12). It has become particularly important in the context of immunotherapy given its inverse relationship with an anti-tumor immune response due, at least in part, to its association with a lymphocyte-excluded phenotype (13). We review the current knowledge of *FGFR3* in the context of both modern therapies such as anti-PD-1 immunotherapy and FGFR blockade.

## *FGFR3* in Bladder Cancer

**Figure 1:**
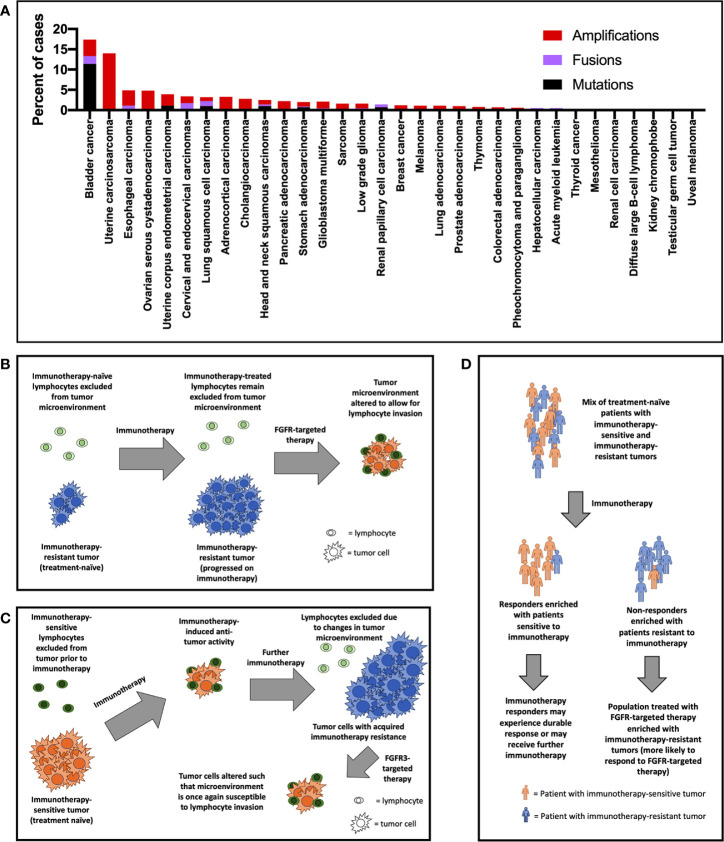
**(A)** *FGFR3* gene alterations by cancer type based on available data from The Cancer Genome Atlas (TCGA) (only recurrent mutations and fusions—those comprising in >1% of mutations/fusions—were included). Potential mechanisms of improved response rate to FGFR3-targeted therapy in the post-immunotherapy setting include **(B)** primary immunotherapy resistance, **(C)** secondary immunotherapy resistance, and **(D)** enrichment of patients with immunotherapy-resistant tumors in trials of FGFR3-targeted therapy.

The chromosome 4 gene *FGFR3* encodes the FGFR3 protein, a tyrosine kinase that has classically been known to play important roles in development, osteogenesis, and bone maintenance (14, 15). *FGFR3* is highly expressed in chondrocytes and osteoblasts, and germline mutations are associated with bone growth disorders such as achondroplasia, chondrodysplasia, and thanatophoric dysplasia (16–20). Curiously, while activating mutations curb growth in bone, the same mutations are associated with excess growth in other tissues (e.g., nevi in skin) (21). Germline *FGFR3* mutations are paternally inherited and are associated with advanced paternal age (22). The introduction of improved clinical genetic testing techniques in oncology has facilitated the discovery that *FGFR3* gene alterations are implicated in a wide range of cancers [**Figure 1A**, (23, 24)]. The prevalence of *FGFR3* gene aberrations is highest in urothelial carcinomas (18% of cases), followed by uterine carcinosarcoma (14%), esophageal (5%), ovarian (5%), and endometrial (4%) cancers (23–25). FGFR3 signaling has been observed to overlap with known oncogenic pathways such as RAS/PI3K/ERK/AKT/EGFR and has been implicated in tumoral epithelial-to-mesenchymal transition (26, 27). The role of *FGFR3* gene in oncogenesis may even be at the pre-translational level: Has_circ_0068871, a circRNA product of *FGFR3* gene transcription, is overexpressed in bladder cancer, and is associated with cancer cell proliferation and migration (28). Expression of the antisense transcript FGFR3-AS1, which increases stabilizes and promotes expression of *FGFR3* mRNA, and which is overexpressed in urothelial tumors, is associated with tumor invasiveness, proliferation, and motility (29). The most common *FGFR3* mutation, S249C, likely develops through an apoprotein B mRNA editing enzyme, catalytic polypeptide-like (APOBEC)-mediated mutagenic mechanism (30). FGFR3-transforming acid coiled coil 3 (TACC3) fusions, which result in constitutive signaling, represent another frequent source of *FGFR3* gene aberration (31).

As prognostic indicators, *FGFR3* gene alterations are generally associated with lower grade and stage among all urothelial bladder carcinomas (32). Among non-muscle invasive cases, 49-84% express *FGFR3*, compared to 18% of muscle-invasive cases, and *FGFR3* mutations are associated with lower disease-specific survival (32–34). Among American Joint Committee on Cancer (AJCC) 8th edition T1 tumors, *FGFR3* expression is associated with lower grade tumor and lower risk of cancer progression (35). *FGFR3* gene mutations, amplifications, and fusions are associated with luminal-papillary subtype of urothelial cancer, which itself is associated with non-muscle invasive disease and favorable prognosis compared with other subtypes (13, 36, 37). However, in spite of the general association of *FGFR3* alterations with favorable characteristics, there is no evidence to suggest that *FGFR3* gene alterations correlate with a less aggressive phenotype once urothelial carcinoma has become advanced. In fact, *FGFR3* gene alterations are associated with less favorable outcomes in the context of chemotherapy for advanced disease (38, 39).

The identification of *FGFR3* as an oncogenic driver in urothelial cancer has led to the development of FGFR3-targeting therapeutics [**Table 1**, (40)]. While the dovitinib, which targets FGFR3, among other tyrosine kinases, showed poor single-agent activity in an unselected urothelial cancer patient population, using pan-FGFR inhibitors with greater target affinity in genomically selected populations has proven to be a more promising approach (41, 42). This observation may reflect a compensation of other FGFR isotypes when therapeutics target FGFR3 on its own. The FGFR1-4 inhibitor erdafitinib is the sole FGFR-targeting agent to which the United States Food and Drug Administration has granted regulatory approval to date. Erdafitinib is indicated for patients with *FGFR2* or *FGFR3*-altered, platinum-treated urothelial cancer (43). Infigratinib, a FGFR1-3 inhibitor, has also demonstrated promising activity (44, 45). Rogaratinib, another pan-FGFR inhibitor is under investigation using FGFR1 or FGFR3 RNA expression levels, rather than genetic mutational status, as a patient selection criterion (46). The most common treatment-emergent toxicities among these agents are hyperphosphatemia, stomatitis, diarrhea, elevated creatinine, fatigue, hand-food syndrome, and decreased appetite. Although the FGFR-inhibitors are undoubtedly becoming a valuable component of the oncologist’s armamentarium for advanced bladder cancer treatment, a greater understanding is needed of how best to combine and sequence these medications with other therapies in the treatment paradigm.

**Table 1:** FGFR inhibitors marketed or in development for bladder cancer.

Medication name	Target	Manufacturer	Phase of development	Patient population	Combination	NCT identifier
**Erdafitinib** (Balversa)	FGFR1-4	Johnson & Johnson	Marketed	FGFR2/3 mutation or fusion	–	NCT02365597
			Ib/II	FGFR2/3 mutation or fusion	Cetrelimab (PD-1 inhibitor)	NCT03473743
**Infigratinib** (BGJ398)	FGFR1-3	BridgeBio Pharma	III	Adjuvant, FGFR3 altered^1^	–	NCT04197986
			Pilot	Non-muscle invasive, FGFR mutation or fusion	–	NCT02657486
**Rogaratinib** (BAY 1163877)	FGFR1-4	Bayer	II/III	high FGFR1 or 3 expression	–	NCT03410693
			Ib/II	cisplatin-ineligible, high FGFR1, or three expression	Atezolizumab (PD-L1 inhibitor)	NCT03473756
**Pemigatinib** (Pemazyre)	FGFR1-3	Incyte	II	FGF or FGFR alteration^2^	–	NCT02872714
			II	platinum ineligible, FGFR3 mutation or rearrangement	Pembrolizumab (PD-1 inhibitor)	NCT04003610
			II	Non-muscle invasive (neoadjuvant)	–	NCT03914794
**Derazantinib** (ARQ 087)	Pan-FGFR	Basilea	Ib/II	FGFR altered^2^	Atezolizumab (PD-L1 inhibitor)	NCT04045613
**Vofatamab** (B701)	FGFR3	Rainier Therapeutics	Ib/II		Pembrolizumab (PD-1 inhibitor)	NCT03123055

^1^“Susceptible” FGFR3 mutations, fusions, or translocations.

^2^Definition of “altered” are not specified.

## FGFR3 as a Therapeutic Target and as a Patient Selection Tool in Context of Immunotherapy for Bladder Cancer

The preclinical and correlative literature underpinning the rationale for combining FGFR3-targeted therapy with immunotherapy is substantial. Research in animal models have contributed to an appreciation of the potential synergies between these two mechanisms. Some studies have suggested that FGFR3 has an important role in regulating the innate immune system, including inhibition of interferons and stimulation of tumor necrosis factor-α (47, 48). Others have noted inhibitory effects on a broad range of components of the adaptive immune response, including lymphocyte infiltration, and T-cell CD8A expression, as well as stimulatory effects on the anti-inflammatory TGF-β response signature (13, 49–52). In fact, our previous work has suggested that *FGFR3* mutations and *FGFR3-TACC3* fusions may be associated exclusively with tumors that exhibit a lymphocyte-excluded phenotype. Moreover, the degree of *FGFR3* expression predicts lymphocyte exclusion (13). Wnt/β-catenin signaling, which is associated with non-T-cell-inflamed tumors both in bladder cancers and across most solid cancers, has been shown to overlap with FGFR3 signaling (13, 53–55). In lung cancer models, FGFR3 inhibition enhances the effect of programmed cell death-1 (PD-1) blockade (56). However, evidence that *FGFR3* pathways work in opposition to immune activity is not uniform: *FGFR3* amplifications are associated with decreased anti-inflammatory M2 macrophage bladder tumor infiltration (51). Additionally, some correlative analyses have not detected a difference in ICI response rates among patients with *FGFR3* mutations compared to those with the wild-type allele (52). Additionally, *FGFR3* mutations are associated with lower PD-L1 expression, a marker that has been shown to have some correlation with ICI response in some bladder cancer trials (7, 50).

Investigational approaches studying the most appropriate role for FGFR inhibition in the context of ICI therapy (either through sequencing or combination) are generally in early clinical stages. The most robust experience available are what appear to be post-hoc analyses of FGFR inhibition following ICI therapy. In erdafitinib’s pivotal trial, patients who had previously received ICI therapy experienced higher response rates compared with the cohort as a whole (59% vs. 40%) (43, 57). Preliminary data with rogaratinib suggest a similar effect: an interim analysis of its phase I trial demonstrated 30% response among ICI-treated patients compared with 24% across all patients (58). There are several potential reasons for the finding of increased responsiveness to FGFR inhibitors after ICI (**Figures 1B–D**). It may be that previous ICI therapy primes patients for FGFR-targeted therapy – i.e., FGFR inhibition “sensitizes” the tumor to the effects of ICI by altering the microenvironment to allow for lymphocyte invasion (**Figure 1B**). Another related explanation for the clinical trial results is that tumors develop enhanced *FGFR3* pathway (lymphocyte exclusionary) signaling as a resistance mechanism while on immunotherapy. Subsequent FGFR inhibition would disrupt this oncogenic tumoral lymphocyte exclusion (**Figure 1C**). A third possibility is that patients who fail immunotherapy tend to be patients whose tumors exhibit poor lymphocyte exclusion (**Figure 1D**). These may be the exact patients who we might expect to benefit most from FGFR-targeted therapy, which may directly address this immune deficit. These may also be patients whose tumors are driven by mechanisms unrelated to the immune system. Importantly, rogaratinib in combination with atezolizumab for first-line urothelial bladder cancer has now shown an objective response rate of 44% including a 16% complete response rate (59). Future research may provide insight to help identify which of these interpretations (or combination of these interpretations or different interpretation altogether) is most accurate. This research may help us understand to what degree FGFR-targeted therapy is best considered as a treatment to be sequenced with immunotherapy. Or, alternatively, to what degree patients who will benefit from FGFR-targeted therapies and those who will benefit from immunotherapy represent two distinct categories. Eventual analyses from currently ongoing phase Ib/II trials testing the FGFR inhibitors vofatamab (NCT03123055), erdafitinib (NCT03473743), and rogaratinib (NCT03473756) in combination with ICI therapies in broad (not genetically selected) populations may enhance our ability to evaluate these propositions.

## Discussion

The *FGFR3* gene is prevalent in bladder cancers and may hold value as a prognostic marker and as a tool for patient selection. *FGFR3* mutations are associated with less aggressive disease across all bladder cancers, although this is not necessarily the case among advanced tumors. Therapies targeting the FGFR3 protein (and its isoforms) have demonstrated clinical benefit in some patients. However, clinicians still require a greater understanding of how these drugs fit into the treatment paradigm alongside immunotherapies. There is conflicting evidence from preclinical and retrospective correlative studies related to the scientific rationale for combining and/or sequencing FGFR-targeted therapies with immunotherapies. To date, the balance of data suggests that there may be a benefit to combining the two types of approaches. However, an alternate theory is that there may be some patients (perhaps those with tumors termed “immune hot” or “lymphocyte invasive”) may be candidates for immunotherapy and not FGFR-targeted therapy, while patients with so-called “immune cold” (or lymphocyte excluded) may be unlikely to benefit from immunotherapy and may be better off with FGFR inhibition earlier on. As FGFR inhibitors become more established in bladder cancer treatment and are studied in earlier lines of therapy, we should gain a more complete view of the best placement of these drugs within therapeutic algorithms.

## Author Contributions

AK and RS conceived and wrote the manuscript. All authors contributed to the article and approved the submitted version.

## Funding

This manuscript was supported by the NIH National Cancer Institute award K08CA234392.

## Conflict of Interest

RS reports consulting/honoraria from Aduro, AstraZeneca, BMS, Exelixis, Eisai, Janssen, Mirati, and Puma. Grant/Research support (to institution): AbbVie, Bayer, BMS, CytomX, Eisai, EpiVax Oncology, Evelo, Genentech, Immunocore, Mirati, Merck, Novartis.

The remaining author declares that the research was conducted in the absence of any commercial or financial relationships that could be construed as a potential conflict of interest.

The handling editor declared a past co-authorship with one of the authors RS.
